# Pazopanib: Approval for Soft-Tissue Sarcoma

**DOI:** 10.6004/jadpro.2013.4.1.6

**Published:** 2013-01-01

**Authors:** Diana T. Nguyen, Sepideh Shayahi

**Affiliations:** From City of Hope Medical Center, Duarte, California

Sarcomas represent a diverse and relatively rare group of solid tumors of mesenchymal cell origin with distinct clinical and histologic features. They can be categorized into two broad groups: sarcomas of soft tissues (including fat, muscles, nerves, nerve sheaths, joints, blood vessels, deep skin tissues, and other connective tissues) and sarcomas of bone (National Comprehensive Cancer Network [NCCN], 2012). Over 50 subtypes of soft-tissue sarcoma (STS) have been identified; most develop in the extremities (60%) yet can also be found in the trunk (19%), retroperitoneum (15%), head and neck (9%), and internal organs (American Cancer Society, 2012; NCCN, 2012). The subtypes vary from relatively indolent to highly aggressive disease and commonly metastasize to the lungs (NCCN, 2012).

An estimated 11,280 new cases and 3,900 deaths from STS occurred in the United States in 2012 (Siegel, Naishadham, & Jemal, 2012). Sarcomas account for about 1% of adult malignancies and 15% of pediatric malignancies (NCCN, 2012). Overall survival (OS) of patients with metastatic STS is about 1 year and has not changed in the past 20 years; thus, new treatment options are needed to improve outcomes (Schöffski, 2012).

Pazopanib (Votrient) is an oral, multitargeted, tyrosine kinase inhibitor (TKI) that was originally approved by the US Food and Drug Administration (FDA) in 2009 for the treatment of advanced renal cell carcinoma (RCC). In April 2012 it gained approval for the treatment of advanced STS in patients who have received prior chemotherapy, excluding those with adipocytic STS or gastrointestinal stromal tumor (GIST) since efficacy in these two patient groups has not been demonstrated. Development of pazopanib for the treatment of STS is discussed in this article; see Table 1 for a summary of key information.

## PHARMACOLOGY AND MECHANISM OF ACTION

Tumor cells require nutrients and oxygen from nearby blood vessels to survive. Without a blood supply, the growth of a solid tumor is limited to approximately 1 to 2 mm3 (Schöffski, 2012). Angiogenesis, the development of new blood vessels, is mediated mainly by vascular endothelial growth factor (VEGF) and also by platelet-derived growth factor (PDGF; Schutz, Choueiri, & Sternberg, 2011). Soft-tissue sarcomas are highly vascularized and produce large amounts of VEGF and VEGF receptors that mediate angiogenesis (Pakos et al., 2005). High tissue VEGF concentration in patients diagnosed with STS has been associated with poor OS and disease progression (Yudoh et al., 2001).

**Table 1:**
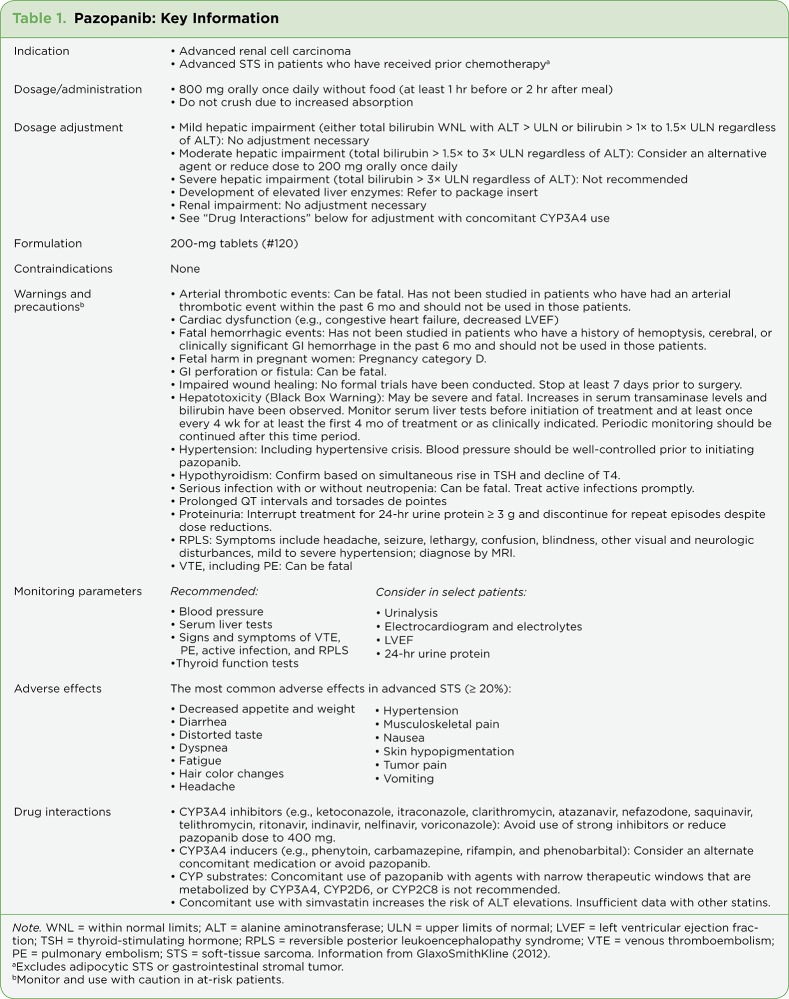
Table 1. Pazopanib: Key Information

As a TKI, pazopanib binds to the VEGF receptor, PDGF receptor, and other proteins found on the cells of blood and lymphatic vessels to inhibit angiogenesis (Schöffski, 2012). This agent is mostly hydrophobic, so it can pass through the double lipid layer of the cell membrane to interact with the intracellular domain of the tyrosine kinase receptor and compete with adenosine triphosphate (ATP) by binding to a target near the ATP-binding cleft of the kinase (Gotink & Verheul, 2010). This inhibits downstream signaling pathways that are involved in angiogenesis, and in doing so, tumor growth and metastasis are inhibited.

## CLINICAL TRIALS

**PHASE I**

The safety and clinical activity of pazopanib were evaluated in a multicentered, dose-finding phase I clinical trial in 63 patients with advanced, refractory solid tumors, including 6 patients with sarcomas. Patients received doses and schedules ranging from 50 mg and 100 mg three times weekly, 50 to 2,000 mg once daily, and 300 mg and 400 mg twice daily.

Four patients experienced dose-limiting toxicities at 50 mg once daily (n = 2, GI hemorrhage and extrapyramidal involuntary movements), 800 mg once daily (n = 1, hypertension and subsequently recurring proteinuria), and 2,000 mg once daily (n = 1, fatigue). A maximum tolerated dose was not determined, but steady-state exposure plateaued at doses ≥ 800 mg once daily; increasing the dose to > 800 mg once daily was not likely to result in consistently greater plasma concentrations with a once-daily schedule. In addition, at 800 mg once daily, 93% of patients achieved a plasma concentration that correlated with hypertension. Thus, 800 mg once daily was chosen for study in phase II trials based on tolerable safety profile, saturation in exposure, and achievement of concentrations that seemed to correlate with clinical activity.

With regard to clinical effectiveness, 17 patients had significant clinical benefit, including 3 with a partial response (2 with RCC and 1 with pancreatic islet cell tumor) and 14 with stable disease for more than 6 months (in various solid tumors; Hurwitz et al., 2009). In this trial, pazopanib was generally well tolerated, with antitumor activity observed across various tumor types, including sarcomas.

**PHASE II**

The safety and efficacy of pazopanib were evaluated in a multicentered, open-label, nonrandomized phase II clinical study in patients with relapsed or refractory high- or intermediate-grade STS who were ineligible for chemotherapy or had received no more than two prior cytotoxic agents sequentially administered as single agents or no more than one prior combination regimen for advanced disease. Patients were treated with pazopanib 800 mg once daily until disease progression, unacceptable toxicity, or withdrawal of consent occurred. The primary endpoint was progression-free rate at 12 weeks (PFR_12 weeks_) after treatment started. This endpoint was used because response rate (RR) does not adequately reflect the antitumor activity of many drugs in STS. A study by Van Glabbeke et al. (2002) supported the use of progression-free rate as the principal endpoint for phase II trials in STS and determined the PFR_12 weeks_ that is associated with active and nonactive agents. Secondary endpoints were progression-free survival (PFS), OS, RR, duration of response, and safety.

The study divided 142 patients into 4 groups: adipocytic sarcomas, leiomyosarcomas, synovial sarcomas, and other eligible STSs. A progression-free rate at 12 weeks that met the predefined criteria of a potentially active agent was found in patients with leiomyosarcomas, synovial sarcomas, and other eligible STSs (44%, 49%, and 39%, respectively). The adipocytic sarcoma group demonstrated insufficient activity (PFR_12 weeks_ of 26%), so accrual was stopped. No complete responses were noted, but partial responses occurred in 9 patients: 1 with leiomyosarcoma, 5 with synovial sarcoma, and 3 with other eligible STSs. Compared with historical controls, PFS and OS were prolonged in the three groups that met the primary endpoint.

The most common adverse effects (AEs) were hypertension (40.1%), fatigue (36.6%), hypopigmentation (36.6%), and nausea (35.9%). Most AEs were grades 1 to 2. The most frequent grades 3/4 toxicities were hyperbilirubinemia (6.3%), hypertension (7.7%), and fatigue (7.7%). For patients who developed hypertension and fatigue, the first incidence usually occurred during the first 4 weeks of treatment. The incidence of diarrhea and hypopigmentation increased with treatment duration (Sleijfer et al., 2009). Based on these findings, further investigation in pazopanib activity in leiomyosarcomas, synovial sarcomas, and other eligible STSs was warranted.

**PHASE III**

PALETTE, a multicentered, international, double-blind, placebo-controlled phase III clinical trial, was the landmark study for pazopanib’s FDA approval for use in STS. The study population included patients with metastatic STS (excluding adipocytic STS and GIST) who had at least one anthracycline-based chemotherapy regimen and had a maximum of four previous lines of systemic therapy (no more than two lines of a combination regimen). Patients (n = 369) were randomized in a 2:1 ratio to either pazopanib 800 mg once daily (n = 246) or placebo (n = 123), and treatment was continued until disease progression, unacceptable toxic effects, withdrawal of consent, or death. The primary endpoint was PFS.

Pazopanib significantly prolonged median PFS (4.6 vs. 1.6 months for placebo; * p* < .0001) across all histologic subtypes that were included. Despite these favorable findings, the difference in OS was not statistically significant (12.5 vs. 10.7 months for placebo; * p* = .25). Clinical benefit was observed in 73% (6% partial response and 67% stable disease) of the pazopanib group, compared with 38% (stable disease only) of the placebo group. Progression occurred in 23% of patients on pazopanib and 57% of patients on placebo. Favorable prognostic factors in patients treated with pazopanib were a good performance status and low or intermediate tumor grade.

The most common AEs were fatigue (65% vs. 49% for placebo), diarrhea (58% vs. 16%), nausea (54% vs. 28%), weight loss (48% vs. 20%), and hypertension (41% vs. 7%). The most common grade 3/4 AEs included fatigue (13%) and hypertension (7%), with rare events of venous thromboembolism, pneumothorax, and decreased left ventricular ejection fraction. The main reasons for dose reductions were hypertension, fatigue, diarrhea, anorexia, nausea and vomiting, hand-foot syndrome, and increased concentration of liver enzymes (Van der Graaf et al., 2012).

## ADVERSE EFFECTS

In a study evaluating the safety of pazopanib in 382 patients with advanced STS, the most commonly observed AEs (≥ 20%) were fatigue, diarrhea, nausea, decreased weight, hypertension, decreased appetite, vomiting, tumor pain, hair color changes, musculoskeletal pain, headache, dysgeusia, dyspnea, and skin hypopigmentation (GlaxoSmithKline, 2012).

Toxicities consistently reported with TKIs are hand-foot skin reaction, hypertension, renal dysfunction, bleeding, fatigue, hypothyroidism, and diarrhea. Nonetheless, differences do exist between pazopanib and other TKIs. For instance, phase II and III trials of pazopanib have shown that the incidence of drug-related rash, hand-foot skin reaction, epistaxis, mouth ulceration, and stomatitis are low and of low grade. Although comparisons between trials of pazopanib, sunitinib (Sutent), and sorafenib (Nexavar) should be interpreted cautiously due to limitations in comparing different populations across trials, pazopanib appears to have a slightly higher incidence of high-grade alanine aminotransferase and aspartate aminotransferase elevation and a lower incidence of myelosuppression, rash, mucositis, hand-foot syndrome, and fatigue/asthenia (Schutz, Choueiri, & Sternberg, 2011). Pazopanib has a black box warning for severe and fatal hepatotoxicity that requires monitoring of hepatic function and interruption, reduction, or discontinuation of dose as recommended by the package insert.

## ROLE IN THERAPY FOR SOFT-TISSUE SARCOMA

For advanced, unresectable, or metastatic STSs, single-agent or combination therapy with an anthracycline or ifosfamide backbone remains the standard of care. Current guidelines from the NCCN recommend pazopanib as an alternative single agent for STSs in the extremity/trunk, head/neck, retroperitoneal, and intra-abdominal regions. It is recommended only for palliative therapy and should not be used for treatment of adipocytic sarcomas (NCCN, 2012). These are category 2A recommendations based on the phase II and III trials reviewed in this article.

## IMPLICATIONS FOR THE ADVANCED PRACTITIONER

Pazopanib is a convenient, well-tolerated, oral treatment option for patients with advanced STS subtypes except for adipocytic sarcomas and GIST. As the utility of pazopanib expands based on findings from clinical trials, it is imperative that the oncology advanced practitioner be familiar with pazopanib. The monthly cost of pazopanib is $7,778.57, which is the average wholesale price of a 120-count bottle of 200-mg tablets (Thomas Reuters, 2012). A patient assistance program is available through GlaxoSmithKline; advanced practitioners can refer patients to www.caresbygsk.com/patients-caregivers.html for details

## SUMMARY

The efficacy of pazopanib in patients with STS who have failed prior chemotherapies provides a new addition to the armamentarium of active drugs in this difficult-to-treat and heterogeneous group of diseases. This TKI has been shown to increase PFS in heavily pretreated patients and is currently recommended as an option for palliative therapy for patients with advanced, unresectable, or metastatic nonadipocytic sarcomas. Information obtained from ongoing studies of pazopanib in the earlier treatment of STS, in combination regimens, and for other cancers will help us understand the unique features of STS subtypes and allow us to select therapeutic approaches using disease-tailored, targeted therapy.
